# Trends in Thermostability Provide Information on the Nature of Substrate, Inhibitor, and Lipid Interactions with Mitochondrial Carriers*

**DOI:** 10.1074/jbc.M114.616607

**Published:** 2015-02-04

**Authors:** Paul G. Crichton, Yang Lee, Jonathan J. Ruprecht, Elizabeth Cerson, Chancievan Thangaratnarajah, Martin S. King, Edmund R. S. Kunji

**Affiliations:** From the Mitochondrial Biology Unit, Medical Research Council, Hills Road, Cambridge CB2 0XY, United Kingdom

**Keywords:** Membrane Protein, Mitochondrial Transport, Protein Stability, Transporter, Uncoupling Protein, Adenine Nucleotide Translocase, Detergent Micelle, Differential Scanning Fluorimetry, Thermofluor Assay

## Abstract

Mitochondrial carriers, including uncoupling proteins, are unstable in detergents, which hampers structural and mechanistic studies. To investigate carrier stability, we have purified ligand-free carriers and assessed their stability with a fluorescence-based thermostability assay that monitors protein unfolding with a thiol-reactive dye. We find that mitochondrial carriers from both mesophilic and thermophilic organisms exhibit poor stability in mild detergents, indicating that instability is inherent to the protein family. Trends in the thermostability of yeast ADP/ATP carrier AAC2 and ovine uncoupling protein UCP1 allow optimal conditions for stability in detergents to be established but also provide mechanistic insights into the interactions of lipids, substrates, and inhibitors with these proteins. Both proteins exhibit similar stability profiles across various detergents, where stability increases with the size of the associated detergent micelle. Detailed analysis shows that lipids stabilize carriers indirectly by increasing the associated detergent micelle size, but cardiolipin stabilizes by direct interactions as well. Cardiolipin reverses destabilizing effects of ADP and bongkrekic acid on AAC2 and enhances large stabilizing effects of carboxyatractyloside, revealing that this lipid interacts in the m-state and possibly other states of the transport cycle, despite being in a dynamic interface. Fatty acid activators destabilize UCP1 in a similar way, which can also be prevented by cardiolipin, indicating that they interact like transport substrates. Our controls show that carriers can be soluble but unfolded in some commonly used detergents, such as the zwitterionic Fos-choline-12, which emphasizes the need for simple validation assays like the one used here.

## Teaser

**Background:** Methods for rapid assessment of interactions of small molecules with membrane proteins in detergent are lacking.

**Results:** Thermostability measurements of mitochondrial transporters display informative trends about detergent, lipid, substrate, and inhibitor interactions.

**Conclusion:** Mechanistic insights are obtained by studying the thermostability of mitochondrial transporters.

**Significance:** Information about the nature of compound interactions with membrane proteins can be obtained rapidly.

## Introduction

Mitochondrial carriers are transport proteins responsible for the exchange of various metabolites across the mitochondrial inner membrane, including nucleotides, vitamins, inorganic ions, and keto and amino acids. They are critical in linking metabolic pathways in several fundamental cellular processes (1). A number of carriers are associated with rare but severe genetic disorders (2), whereas uncoupling proteins, which are also members of this protein family, may be important in our understanding and treatment of obesity and the metabolic syndrome (3, 4).

Mitochondrial carriers are composed of three ∼100-amino acid homologous domains, each comprising two transmembrane α-helices separated by a matrix loop and small α-helix (5–7). The first helix of each domain contains the signature motif P*X*(D/E)*XX*(R/K), which is well conserved across the protein family (5). Much of our structural and mechanistic understanding of mitochondrial carriers has come from studies on the ADP/ATP carrier (see Ref. 8 and references therein), which can be fixed in distinct states by specific high affinity inhibitors. Carboxyatractyloside (CATR)^3^ (9) and bongkrekic acid (10) lock the carrier in a conformation with the substrate-binding site facing the intermembrane space/cytosol (c-state) or the mitochondrial matrix (m-state), respectively. A projection map in the membrane (11) and several atomic structures of the bovine and yeast ADP/ATP carriers in detergent (7, 12, 13), stabilized by CATR, are now available and show a consistent carrier fold. The six transmembrane helices from the three homologous domains form a barrel arrangement with 3-fold pseudosymmetry, where the charged residues of the P*X*(D/E)*XX*(R/K) motif from each domain contribute to a salt bridge network that closes a central cavity on the matrix side (7, 14).

Mitochondrial carriers function as monomers (see Ref. 15), and several lines of evidence have highlighted key aspects within the structural fold that are important for substrate exchange. Distance and chemical constraints (16), symmetry analysis (17), molecular dynamics simulations (18, 19), and mutagenesis (20) are consistent with a single substrate-binding site being present in a central water-filled cavity. Charge reversal mutagenesis has confirmed that a second conserved motif, (Y/F)(D/E)*XX*(K/R), identified in the second transmembrane helix of each domain (17), forms an additional salt bridge network toward the cytoplasmic side of carriers during the transport cycle (13). These features, along with the domain arrangement, are consistent with a domain-based alternating access mechanism, where matrix and cytoplasmic salt bridge networks gate the access of substrates to the central substrate-binding site (13, 17). The uncoupling proteins have the same structural fold and sequence features described above, but to what extent they utilize a common carrier exchange process is not known. Uncoupling protein 1 (UCP1), responsible for thermogenesis in brown adipose tissue, catalyzes the fatty acid-dependent, purine nucleotide-sensitive transport of protons across the mitochondrial inner membrane by a disputed mechanism (21).

Mitochondrial carriers have been found to be unstable when purified in various detergents, which hampers structural and mechanistic investigations. The ADP/ATP carrier requires stabilization with CATR to allow purification in mild non-ionic detergents (22–25). The purification yields of many ligand-free carriers are generally poor but can be improved in the presence of cardiolipin (see Refs. 26 and 27 and references therein). This lipid is well known to influence carrier functioning (26, 27) and can be observed in the atomic structures of the CATR-ADP/ATP carrier complex (7, 12, 13). Three cardiolipin molecules bind to the surface of the protein, arranged in accordance with the 3-fold pseudosymmetry, where each lipid headgroup interacts with two domains on the matrix side of the protein (13). An exception is UCP1, which is reported not to bind cardiolipin tightly (27) and appears to be more stable than other carriers during purification (28). Even so, the protein still requires mild conditions during purification because it loses function in zwitterionic and ionic detergents (28, 29). Observations relating to carrier stability are generally consistent across many studies, although some, particularly those with *Escherichia coli* expressed carriers, do not fit general trends. Bacterially expressed UCP1 and UCP2 have been reported to be stable in the relatively harsh zwitterionic detergents *N*,*N*-dimethyldodecylamine-*N*-oxide (30) and Fos-choline-12 (Fos12 or DPC) (30, 31), where the physiological relevance of UCP2 preparations has been questioned (29). Robust methods are required, therefore, to assess the stability of carrier proteins and verify native-like folded states.

To characterize the stability of mitochondrial carriers, we have adapted a protein thermostability assay that utilizes the thiol-reactive fluorophore *N*-[4-(7-diethylamino-4-methyl-3-coumarinyl)phenyl]maleimide (CPM) (32) for use with a rotary qPCR multisample instrument. Despite using non-ideal fluorescent wavelength channels, the relative stability of membrane protein samples can be assessed rapidly with microgram amounts of protein. We present trends observed in the stability of mitochondrial carriers, focusing on yeast AAC2 and ovine UCP1, which provide mechanistic information on the interaction of ligands and lipids with these proteins.

## MATERIALS AND METHODS

### 

#### 

##### Expression of Carriers in Yeast

Gene sequences for each ADP/ATP carrier with an N-terminal eight-histidine tag and Factor Xa cleavage site (codon optimized for *Saccharomyces cerevisiae* expression by Genscript) were cloned into a modified pYES3 vector under the control of the promoter for the *S. cerevisiae* phosphate carrier *PIC2*, as described previously for yeast AAC2 (13). The sequences used encode MtAAC1 residues 8–308 (XP_003667288 from *Myceliophthora thermophila*), TlAAC1 residues 10–311 (Thela2p4_002753 from *Thermomyces lanuginosus*; see the Genozymes Web site), CmAAC1 residues 19–322 (XP_005537244 from *Cyanidioschyzon merolae*), and GsAAC1 residues 80–381 (XP_005704429 from *Galdieria sulfuraria*). The genes were confirmed by sequencing (Source Bioscience), and the expression vectors were transformed into *S. cerevisiae* strain WB12 (MATα *ade2-1 trp1-1 ura3-1 can1-100 aac1*::*LEU2 aac2*::*HIS3*) (33), which lacks functional Aac1p and Aac2p carriers. Transformants were first selected on SC medium minus Trp plates (Invitrogen) and then on YEPG plates to show that the expressed ADP/ATP carrier was functional and rescued growth on a non-fermentable carbon source (yeast expressing MtAAC1, TlAAC1, CmAAC1, and GsACC1 exhibited growth rates of 0.13, 0.15, 0.08, and 0.03 h^−1^, respectively, in liquid cultures). Precultures were used to inoculate 80–100 liters of YPG in a bioreactor set up, and cells were harvested as described previously (13).

For the expression of human citrin, the codon-optimized gene sequence (Q9UJS0) with an N-terminal eight-histidine tag and tobacco etch virus cleavage site was introduced into a pYES3/CT vector (Invitrogen) under the control of the galactose-inducible promoter *GAL1*, as described previously (34). The vector was transformed into *S. cerevisiae* strain W303-1B, and transformants were selected on SC medium minus Trp plates. Large scale cultures (100 liters) were grown in YPG medium containing 0.1% glucose in a bioreactor setup, protein expression was induced, and cells were harvested, as described previously (34).

##### Preparation of Mitochondria and Protein Purification

Yeast mitochondria were isolated following cell disruption using a bead mill, as described previously (13). Brown adipose tissue was extracted from newborn lambs that had died of natural causes (from local farms), and mitochondria were isolated using established methods (35). Tissue and mitochondrial samples were flash frozen and stored in liquid nitrogen, as required.

His-tagged ligand-free ADP/ATP carriers were purified by nickel affinity chromatography based on a procedure described previously (36). 0.25–1.0 g of yeast mitochondria were solubilized in a 2% dodecyl-β-d-maltoside (12M) or undecyl-β-d-maltoside (11M; for yeast AAC2) solution for 30 min at 4 °C containing 150 mm NaCl, 20 mm imidazole, 10 mm Tris, pH 7.4, and two tablets of Complete protease inhibitor minus EDTA per 100 ml (Roche Applied Science). Insoluble material was removed by centrifugation (140,000 × *g* for 20 min, 4 °C), and the supernatant was loaded onto a nickel-Sepharose column (high performance; GE Healthcare) at 1 ml/min using an ÄKTAprime FPLC system. The column was washed at 3 ml/min with 50 column volumes of buffer A (containing 150 mm NaCl, 60 mm imidazole, 10 mm Tris, pH 7.4, with 0.1% 12M (or 0.1% 11M for yeast AAC2), and 0.1 mg/ml tetraoleoyl cardiolipin included) followed by 30 column volumes of buffer B (containing 50 mm NaCl, 10 mm Tris, pH 7.4, and detergent and lipid as in buffer A). To cleave the protein from the column, the nickel-Sepharose was recovered as a slurry (∼1.2 ml) and treated with factor Xa protease (with 5 mm CaCl_2_; New England Biolabs) either overnight at 10 °C (120 units) or, for yeast AAC2, for 3 h at 4 °C (40 unit) in the presence of 20 mm imidazole. The slurry was transferred to an empty Micro Bio-Spin column (Bio-Rad) and centrifuged (500 × *g*, 5 min at 4 °C) to separate the protein from the resin. Residual nickel-Sepharose contamination was removed by another centrifugation step (12,000 × *g*, 10 min at 4 °C) in a 2-ml tube. For yeast AAC2 preparations, samples were also spun through a Vivapure Q Mini H spin column (Sartorius). The final purified protein was quantified by BCA protein assay (Thermo Scientific) with bovine serum albumin as a standard.

His-tagged citrin was purified as described previously (34) with some modifications. Yeast mitochondria were solubilized in 2% 12M solution containing 150 mm NaCl, 20 mm imidazole, 20 mm Hepes, pH 8.0, with Complete protease inhibitor minus EDTA. Insoluble material was removed by centrifugation, and the supernatant was loaded onto a nickel-Sepharose column as described above. The column was washed with 50 column volumes of buffer A (300 mm NaCl, 60 mm imidazole, 20 mm Hepes, pH 8.0, with 0.1% 12M and 0.1 mg/ml tetraoleoyl cardiolipin included) followed by 20 column volumes buffer B (150 mm NaCl, 20 mm Hepes, pH 8.0, with 0.05% 12M and 0.05 mg/ml tetraoleoyl cardiolipin included). Citrin was cleaved from the resin by overnight treatment with 540 μg of maltose-binding protein-tagged tobacco etch virus protease in the presence of 60 mm imidazole and 1 mm dithiothreitol. The cleaved protein was separated from the Sepharose using a Proteus Midi Spin column (Generon), concentrated with a 100,000 molecular weight cut-off concentrator (Sartorius), and loaded onto a Superose 6 10/300 GL size exclusion column equilibrated with buffer (150 mm NaCl, 20 mm Hepes, pH 8.0, with 0.03% 12M and 0.03 mg/ml tetraoleoyl cardiolipin included). Peak fractions were collected and concentrated to 1 mg/ml protein (BCA assay) before storage in liquid nitrogen.

Ovine UCP1 was purified by new methods.^4^ Brown adipose tissue mitochondria were stirred in alkali buffer (100 mm Na_2_CO_3_, pH 11.5, 1 mm EDTA) for 30 min (4 °C) to lyse mitochondria and strip the membranes of peripherally associated proteins. The membranes were harvested by centrifugation (200,000 × *g* for 40 min) and resuspended in wash buffer (20 mm Tris, pH 7.4, 1 mm EDTA, 10% glycerol), followed by a repeat centrifugation and resuspension in wash buffer (without EDTA) before flash freezing in liquid nitrogen for storage. 50–60 mg of enriched membranes were thawed, collected by centrifugation, and resuspended in solubilization buffer (3–4% decyl maltose neopentyl glycol (10MNG), 300 mm NaCl, 20 mm Tris, pH 8.0, with Complete protease inhibitor minus EDTA) to ∼10 mg/ml protein. The sample was stirred for 1 h (<10 °C) and centrifuged (250,000 × *g* for 20 min) to remove insoluble material, and the supernatant was collected and desalted using PD-10 columns (GE Healthcare). UCP1 was purified by passage through a Vivaspin S Maxi H spin column (Sartorius), supplemented with 50 mm NaCl, and further purified by passage through a Vivapure Q Maxi H spin column. To allow detergent exchange and the removal of excess detergent and lipid, the protein was immobilized by covalent chromatography. The purified preparation was supplemented with 150 mm NaCl, 1 mm EDTA and 50 mm Tris, pH 8.0, and mixed with thiopropyl-Sepharose 6B (Sigma; 100–150 mg dry/mg of UCP1, prerinsed in deoxygenated water) for 1 h in an empty PD-10 column (<10 °C). The column was packed by gravity flow (the eluate discarded) and washed with 100 ml of deoxygenated TPS buffer (50 mm Tris, pH 8.0, 150 mm NaCl, 1 mm EDTA, with 0.05% 10MNG or 0.02% 12M with or without 0.05 mg/ml tetraoleoyl cardiolipin) at a flow rate of ∼5 ml/min. To recover UCP1 from the material, the damp resin was mixed with 2.5 ml of TPS buffer containing 150 mm dithiothreitol for 15 min (<10 °C), and the column was centrifuged (500 × *g*, 5 min). The eluate containing pure UCP1 was exchanged into storage buffer (either 10 mm Tris, pH 7.4, 0.05% 10MNG or 10 mm Tris pH 8, 0.02% 12M) using a PD-10 desalting column and concentrated to ∼5 mg/ml before storage in liquid nitrogen.

Mouse UCP1 was expressed in *E. coli* as inclusion bodies as described in (37) and purified as detailed in (38). The purified inclusion body pellets were solubilized in a 5% Fos12 solution (containing 30 mm sodium phosphate, pH 6.5, and 80 mm NaCl) and mixed for 1 h (4 °C) before the insoluble material was removed by centrifugation (16,000 × *g* for 20 min). The final preparation contained 5 mg/ml protein (BCA assay).

##### Protein Thermostability Assay

A fluorescence-based procedure for the assessment of membrane protein stability (see Alexandrov *et al.* (32)) was adapted for high throughput use on a rotary qPCR instrument. In preparation for use, 5 mg/ml stocks of CPM dissolved in DMSO (thawed from −80 °C storage on the day) were diluted 50-fold into the desired assay solution (20 mm Hepes, pH 7.5, 50 mm NaCl, unless stated otherwise, with detergent as indicated) and incubated for 10 min at room temperature. For each test, purified carrier protein (typically 1.5–2.0 μg) was diluted to 45 μl in the same assay solution and incubated on ice for ≥10 min in 200-μl thin-walled PCR tubes. 5 μl of the CPM dilution was added, and the sample was incubated on ice for a further 15 min with occasional mixing. The samples were subjected to a high resolution melt (HRM) procedure on a Rotor-Gene Q 2plex HRM qPCR cycler with a 36-sample rotor (Qiagen). Note that a lower assay volume could also be used with a 72-sample rotor. The instrument software was set to increase the temperature from 25 to 90 °C in 1 °C increments with the “wait between reading” set to 4 s, which equated to a temperature ramp of 5.6 °C/min. The gain setting was operated at 0 or 0.33, which resulted in fluorescent signals in a suitable detection range of the instrument. In accordance with the software, the first 90 s of the program was used to allow the instrument to reach an equilibration temperature (seven increments below the start temperature; 18 °C) and then rise to the start temperature of the ramp, giving an overall program time of <15 min. During runs, the rotary function of the machine ensured a uniform temperature across all samples and counteracted problematic foaming associated with detergent. Importantly, the machine was allowed to cool to 18 °C (the ambient temperature) between runs. In the assay, buried cysteine residues become solvent-exposed as protein unfolds and react with CPM to form a fluorescent adduct (excitation/emission optima of 387/463 nm). The high brightness of the excitation light on the HRM channel (which provides maximal excitation light at 440–480 nm, with emission detected at 505–515 nm) allowed adduct formation to be monitored at non-optimal wavelengths. Importantly, control tests on a conventional fluorometer showed that any changes in the signal strength of CPM-reacted carrier protein induced by detergent, lipid, or changes in temperature were proportional across the whole excitation or emission wavelength spectra (data not shown). Protein unfolding profiles were analyzed using the Rotor-gene Q software, and the peak in the derivative of the fluorescence signal as a function of temperature, the “melt” temperature (*T_m_*), provided a relative measure of protein stability.

##### Proteoliposome Reconstitution and Proton Flux Assays

The reconstitution of UCP1 and measurement of proton uptake activity were carried out following the methods of Echtay *et al.* (39). 20 μg of Fos12-solubilized UCP1 inclusion body material or native UCP1 in 10MNG was reconstituted into phosphatidylcholine (18:1) liposomes loaded with 100 mm K^+^ (potassium phosphate, pH 7.5) and 0.2 mm EDTA and exchanged into external buffer (110 mm sucrose, 1 mm K^+^ (gluconate), 0.5 mm Hepes, pH 7.5). Proton uptake was measured by following the pH-sensitive fluorescence of external pyranine (λ_ex_ = 467 nm, λ_em_ = 510 nm) at 25 °C. 75 μl of proteoliposomes were diluted to 500 μl in external buffer (pH 8.2) containing 1 μm pyranine, 200 μm oleic acid (1.6 mm methyl-β-cyclodextrin), and 40 μm GDP where indicated. The fluorescent signal of the sample was adjusted to pH 7.5 with 7.5 mm H_2_SO_4_ in 30-nmol H^+^ steps to calibrate the signal/proton change. A membrane potential was induced by the addition of 2.5 μm valinomycin to drive proton uptake, and the total proton uptake capacity of the system was revealed through the subsequent addition of 1 μm carbonyl cyanide *p*-chlorophenylhydrazone. Initial rates of proton uptake were estimated from fits of the valinomycin-induced progress curve using an appropriate exponential function (“plateau and one phase association”; GraphPad Prism software).

## RESULTS

For the rapid and economical assessment of membrane protein stability, we have adapted a CPM thermofluor assay (see Alexandrov *et al.* (32)) for use with a qPCR multisample instrument (Rotor-Gene Q 2plex HRM cycler). In the assay, the temperature of protein samples is increased from 25 to 90 °C while protein unfolding is monitored with CPM. The compound reacts with protein thiols as they become solvent-exposed due to denaturation to give a fluorescent adduct. We have found that, despite using non-ideal excitation and emission wavelength channels for CPM (see “Materials and Methods”), the high brightness of the HRM channel on the Rotor-Gene Q instrument allowed unfolding to be monitored reliably with as little as 1.5 μg of protein. Furthermore, the accompanying software, designed for double-stranded DNA melt analysis, provided a rapid and convenient tool to estimate protein “melt” temperatures.

### 

#### 

##### Mitochondrial Carriers Are Inherently Unstable

**FIGURE 1.**
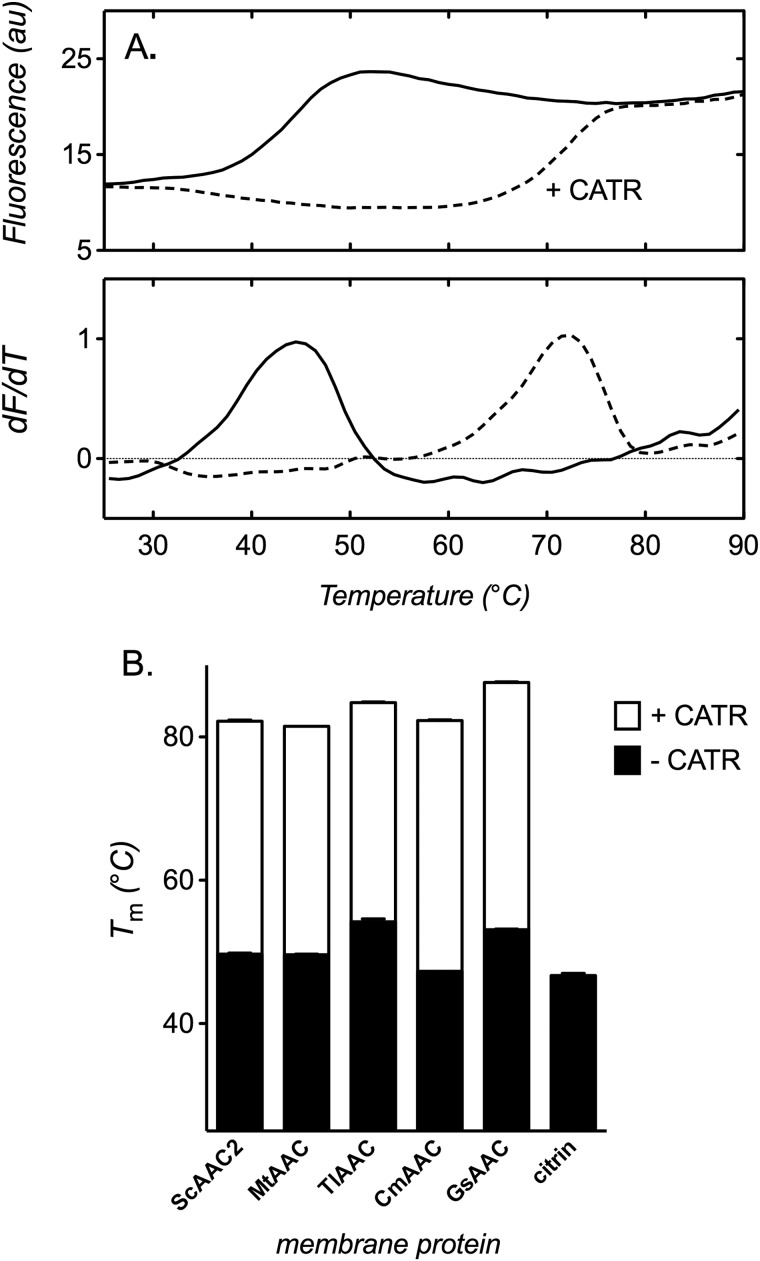
**The relative thermal stability of mitochondrial carrier proteins.** Carrier unfolding was monitored by the fluorescence of CPM-adduct formation at cysteine residues as they become solvent-exposed due to thermal denaturation (see “Materials and Methods”). *A*, thermal denaturation profile (*top*), and corresponding first derivative (*bottom*), of yeast AAC2 in assay buffer with 1.0% 12M in the absence (*solid line*) or presence (*dashed line*) of 20 μm CATR. *B*, apparent *T_m_* of several AAC isoforms and human citrin in purification buffer (0.1% 12M, 50 mm NaCl, 10 mm Tris, pH 7.4, with 0.1 mg/ml tetraoleoyl cardiolipin) in the absence or presence of 20 μm CATR, as indicated. Typical profiles are shown, and *T_m_* values are averages ± S.D. (*error bars*) of three tests. ScAAC2 (4 cysteines), MtAAC1 (2 cysteines), TlAAC (3 cysteines), CmAAC (2 cysteines), GsAAC (4 cysteines) and citrin (7 cysteines) are from *S. cerevisiae*, *M. thermophila*, *T. lanuginosus*, *C. merolae*, *G. sulfuraria*, and *Homo sapiens*, respectively.

To investigate carrier stability, we purified several mitochondrial carrier proteins in mild detergents, stabilizing them with cardiolipin in the purification buffers (see “Materials and Methods”). Small amounts of the protein were exchanged into the appropriate assay buffer by dilution and incubated before stability measurements were made to allow the sample components to equilibrate. When assessed, the carriers gave typical sigmoidal profiles, with an initial low signal plateau that transitions to a higher plateau with an increase in temperature (*e.g.* yeast AAC2; Fig. 1*A*, *top*), consistent with the unfolding of the protein and exposure of internal cysteine residues to CPM. The unfolded protein signal is proportional to the number of thiols detected in the assay but decreases mildly across the profile, reflecting a decrease in quantum yield of the fluorophore with temperature (also see Ref. 32). The peak in the derivative of each profile (*e.g.* yeast AAC2; Fig. 1*A*, *bottom*) was used to determine a *T_m_* value, to provide a relative measure of protein stability.

In assay buffer with mild detergent (1% 12M), yeast AAC2 exhibited an apparent *T_m_* of 44.7 ± 0.3 °C (Fig. 1*A*). This value is low but could be significantly increased to 72.2 ± 0.1 °C by CATR (Fig. 1*A*), consistent with the typical effect of ligands on protein stability (40). Several AAC isoforms from thermophilic fungi (*M. thermophila* and *T. lanuginosus*) and red algae (*C. merolae* and *G. sulfuraria*) were also expressed in yeast mitochondria, purified, and assessed. When tested alongside yeast AAC2 in purification buffer, containing 0.1% 12M with 0.1 g of cardiolipin/g of detergent, the stability values for these proteins were at maximum only 5 °C higher than yeast AAC2 (with or without CATR; Fig. 1*B*), despite the thermophilic organisms growing at temperatures up to 20 °C higher than mesophilic yeasts (41–43). In addition, ovine UCP1 (see data below) and the human aspartate/glutamate carrier citrin, which has an EF-hand Ca^2+^-binding domain in addition to the common carrier fold, also exhibited a *T_m_* similar to that of yeast AAC2 (46.7 ± 0.3 °C; Fig. 1*B*). Although not exhaustive, the consistently low *T_m_* values observed here would suggest that instability is an inherent property of mitochondrial carrier proteins in detergent.

##### AAC2 Stability Increases with the Size of the Detergent Micelle

**FIGURE 2.**
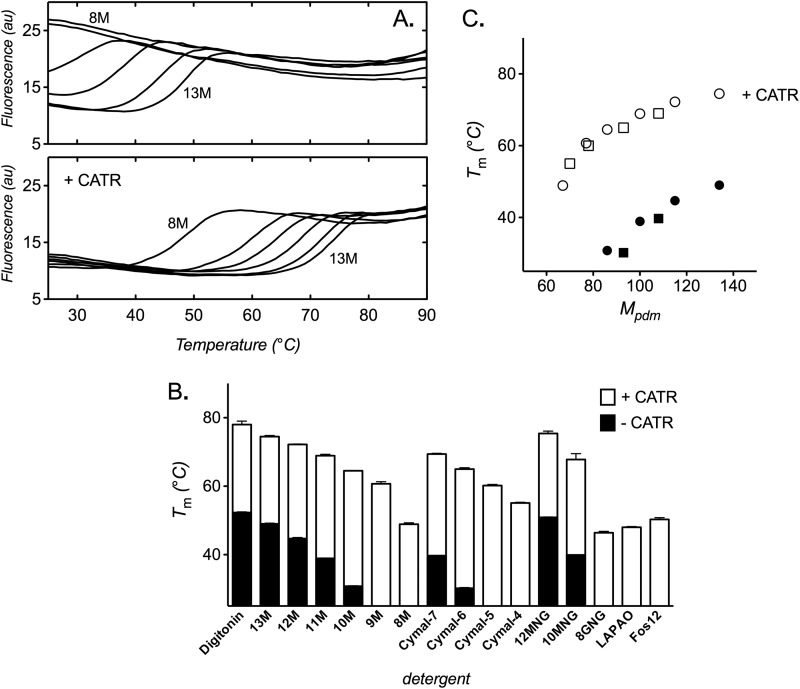
**The influence of detergent on the thermal stability of yeast AAC2.** Protein stability assays were performed as described under “Materials and Methods” with 1% detergent (2% for 8M) in the assay buffer. *A*, thermal denaturation profiles of yeast AAC2 in alkyl maltoside detergent (8M–13M, *left* to *right*) in the absence (*top*) or presence (*bottom*) of 20 μm CATR. *B*, apparent *T_m_* of yeast AAC2 in various detergents in the absence or presence of 20 μm CATR, as indicated. *C*, dependence of AAC2 stability on the estimated mass of the protein-detergent micelle (*M_pdm_*) in the alkyl maltoside (*circles*) or Cymal (*squares*) detergent series, in the absence (*closed symbols*) or presence (*open symbols*) of CATR (*M_pdm_* values were taken from Refs. 24 and 25 for yeast AAC3). Typical profiles are shown, and *T_m_* values are averages ± S.D. (*error bars*) of three tests. *8GNG*, octyl glucose neopentyl glycol.

We determined the relative stability of AAC2 in various detergents, both in the absence and presence of CATR (Fig. 2). In the alkyl maltoside series of detergents, the stability of AAC2 varied with the length of the detergent alkyl chain, with a *T_m_* value of 49.0 ± 0.2 °C in 13M that decreased to below 25 **°**C (the lower limit of the assay) in 9M and 8M (Fig. 2, *A* (*top*) and *B*). A similar trend occurred in the presence of CATR but with unfolding transitions at significantly higher temperatures (*T_m_* values of 74.5 ± 0.3 to 48.9 ± 0.4 °C in 13M to 8M, respectively, Fig. 2, *A* (*bottom*) and *B*). In the Cymal series of detergents, essentially the same patterns were observed (see Fig. 2*B*). These trends relate to the size of the detergent micelle associated with the carrier, which varies with the size of the detergent alkyl chain length (24, 25). When expressed as a function of the protein-detergent micelle mass for a carrier in detergent (*M_pdm_*; determined previously for yeast AAC3 (24, 25)), AAC2 stability showed overlying trends in the maltoside and Cymal detergent series (Fig. 2*C*). In the absence of CATR, stability increased with *M_pdm_* but less so toward higher *M_pdm_* values. A similar but milder trend was also apparent in the presence of CATR at generally higher stability values, with smaller changes in *T_m_* associated with CATR binding at higher *M_pdm_* values. The detergent micelle size would therefore appear to be a dominant factor in determining carrier stability. Consistent with this, AAC2 showed the highest *T_m_* values in digitonin (Fig. 2*B*), which has the largest micelle size (24). However, other properties of detergents must be important too. In the zwitterionic detergents, LAPAO and Fos12, the stability of AAC2 was too low to be determined (a *T_m_* of <25 °C) unless the carrier was stabilized by CATR (Fig. 2*B*). This was also the case in the conjugated detergent octyl glucose neopentyl glycol (8GNG). However, in the larger detergents of this type, decyl- and dodecyl-maltose neopentyl glycol (10MNG and 12MNG, respectively), AAC2 stability increased with detergent size, to give *T_m_* values even higher than those observed in the non-conjugated detergent equivalents, 10M and 12M (see Fig. 2*B*).

##### Lipids Stabilize AAC2 by Different Mechanisms

**FIGURE 3.**
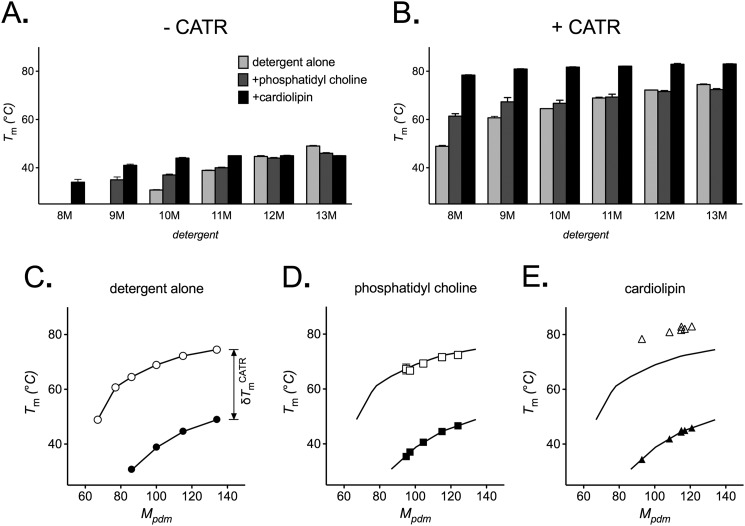
**The influence of lipids on the thermal stability of yeast AAC2.** Apparent *T_m_* values for AAC2 were determined (see “Materials and Methods”) in 1% 9M–13M or 2% 8M, with 14:0 phosphatidylcholine or cardiolipin (0.1 g/g of detergent) present as indicated, in the absence (*A*) or presence (*B*) of 20 μm CATR. *C*, the *T_m_* values for AAC2 in alkyl maltoside detergent alone as a function of the estimated mass of the protein-detergent micelle (*M_pdm_*) in the absence (*closed circles*) or presence (*open circles*) of CATR (as shown in Fig. 2*C*). *D* and *E*, *T_m_* values for AAC2 obtained in the presence of lipid (from *A*) fitted onto the trend line observed in *C* (−*CATR*) to give *M_pdm_* values that were also used to plot the corresponding *T_m_* values obtained in the presence CATR (from *B*). Only in the presence of phosphatidylcholine (*D*), but not cardiolipin (*E*), do the corresponding *T_m_* values fit the second trend line from C (+*CATR*). See “Results.” *T_m_* values are averages ± S.D. (*error bars*) of three tests.

We explored the influence of lipids on the stability of AAC2 in the maltoside series of detergents. Both phosphatidylcholine and cardiolipin increased the stability of AAC2 in the smaller detergents of the series, where the stability had originally been lower, but had progressively less effect as the detergent size increased, with little or no effect in 12M and 13M (Fig. 3*A*). Cardiolipin induced larger stabilizing effects than phosphatidylcholine (*e.g.* in 10M, the *T_m_* increased by 13.2 and 6.2 °C, respectively; Fig. 3*A*), although AAC2 stability with both lipids tended toward the same maximum *T_m_* value (∼45 °C) with increasing detergent size. The preferential effect of lipids in smaller detergents would suggest that they work indirectly by increasing the effective size of the detergent micelle. In the presence of CATR, similar trends were observed with phosphatidylcholine at generally higher stability values, with little effect of the lipid in the larger detergents (Fig. 3*B*). In contrast, cardiolipin increased AAC2 stability to a greater extent, even in the larger detergents, to give *T_m_* values of ∼80 °C across the whole detergent series (Fig. 3*B*).

In the absence of added lipid in the alkyl maltoside series of detergents, the stability of AAC2 exhibited particular trends with *M_pdm_*, where the change in *T_m_* value associated with CATR (δ*T_m_*^CATR^) also varied with *M_pdm_* (decreasing from 33.7 °C at 86 kDa to 25.5 °C at 134 kDa in 10M–13M; Fig. 3*C*). We hypothesized that, in the presence of lipids, AAC2 stability should follow these same relationships if lipids increase stability by increasing the effective detergent micelle size. Accordingly, a given stability value obtained in the absence of CATR should fit the observed trends at a particular *M_pdm_* where the corresponding stability value obtained in the presence of CATR also fits the trends (*i.e.* with a particular value of δ*T_m_*^CATR^). For AAC2 in phosphatidylcholine, this is indeed the case. The *T_m_* values obtained in the absence of CATR predict particular *M_pdm_* values where the corresponding *T_m_* values obtained in the presence of CATR fitted the appropriate trend line well (Fig. 3*D*). However, for AAC2 in cardiolipin, the corresponding *T_m_* values did not fit the same trend line well, falling at least 10 °C higher (Fig. 3*E*). These trends suggest that phosphatidylcholine stabilizes AAC2 purely by increasing the effective detergent micelle size, whereas cardiolipin stabilizes the CATR-bound state, at least in part, by an additional mechanism.

##### Cardiolipin Reverses a Destabilizing Effect of Substrate and Bongkrekic Acid on AAC2

**FIGURE 4.**
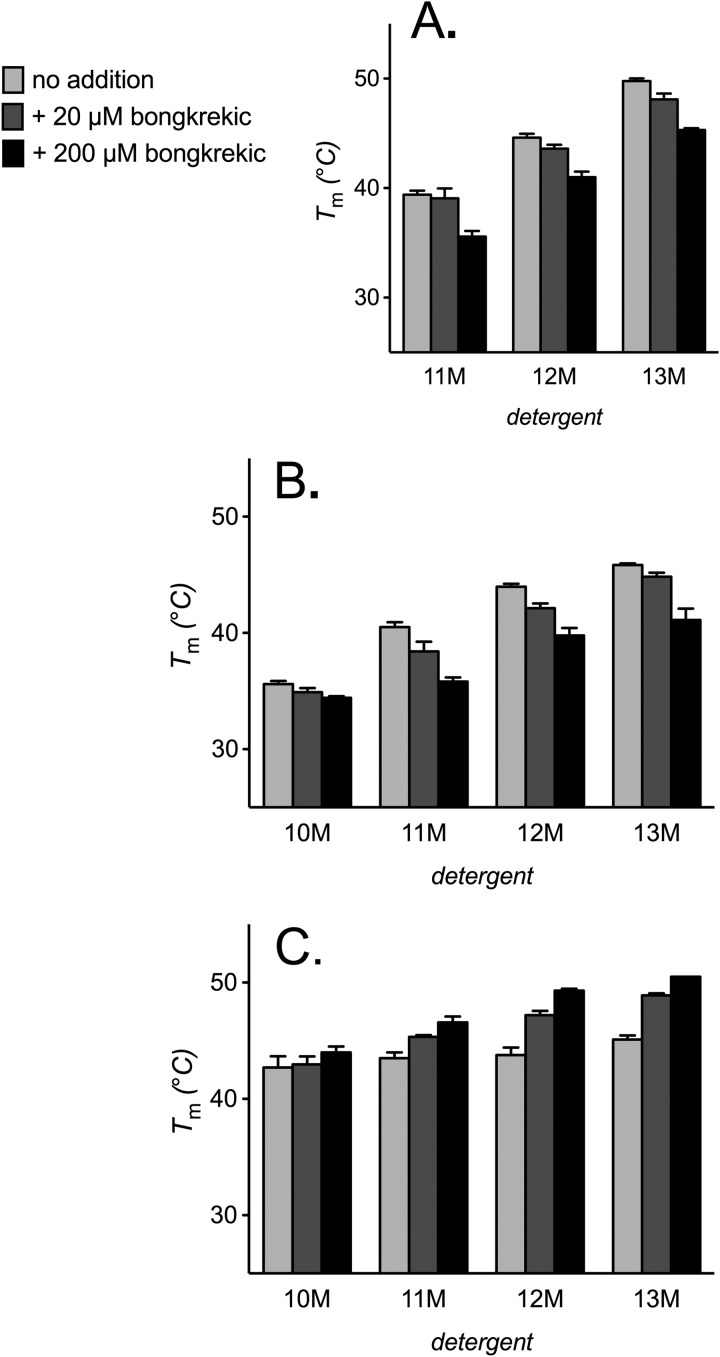
**The influence of lipids on bongkrekic acid-induced changes in AAC2 thermal stability.** Apparent *T_m_* for AAC2 were determined (see “Materials and Methods”) in 1% alkyl maltoside detergent alone (*A*) or with 14:0 phosphatidylcholine or cardiolipin (0.1 g/g of detergent) present (*B* and *C*, respectively) with 20 or 200 μm bongkrekic acid present where indicated. *T_m_* values are averages ± S.D. (*error bars*) of three tests.

**FIGURE 5.**
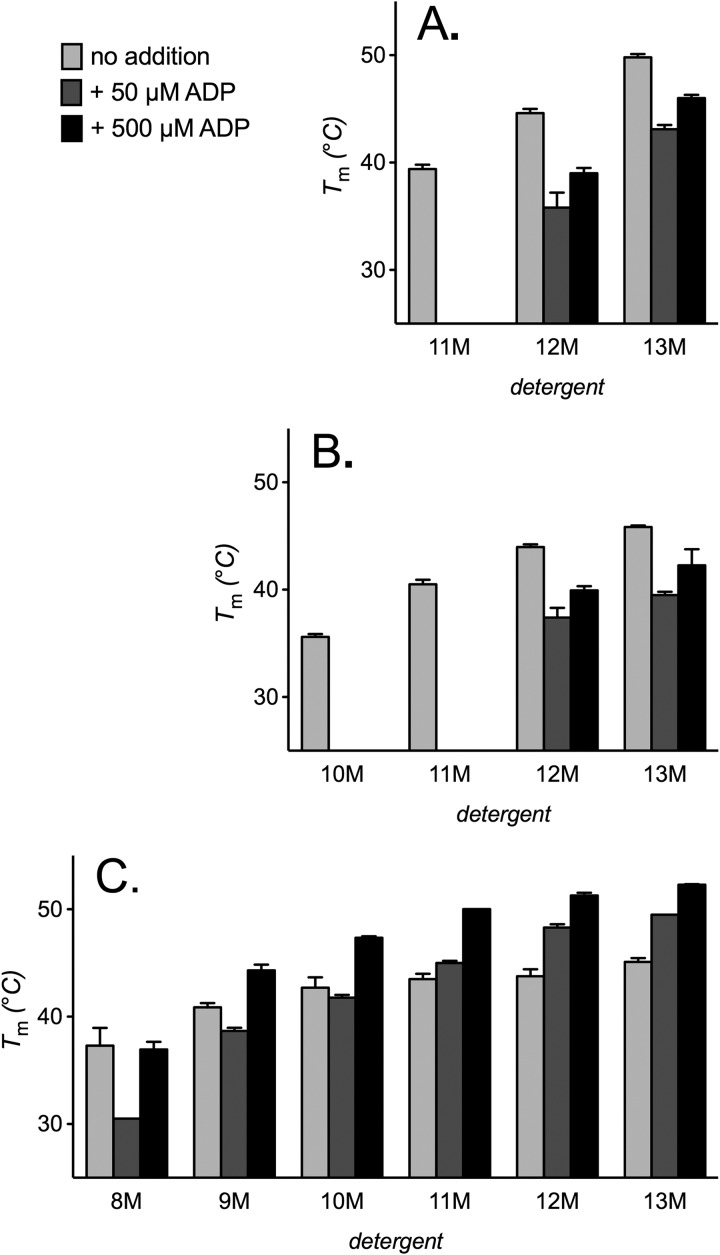
**Influence of lipids on ADP-induced changes in AAC2 thermal stability.** Apparent *T_m_* values for AAC2 were determined (see “Materials and Methods”) in 1% 9M–13M or 2% 8M detergent alone (*A*) or with 14:0 phosphatidylcholine or cardiolipin (0.1 g/g of detergent) present (*B* and *C*, respectively) with 50 or 500 μm ADP present where indicated. *T_m_* values are averages ± S.D. (*error bars*) of three tests.

In the alkyl maltoside detergents, the inhibitor bongkrekic acid induced a mild concentration-dependent destabilization of AAC2, with up to a ∼5 °C decrease in *T_m_* (Fig. 4*A*). Ligand binding is expected, in general, to increase protein stability (see Ref. 40), although this effect would be consistent with a conformational change in the carrier to a less stable state. In the presence of cardiolipin (but not phosphatidylcholine), bongkrekic acid induced the opposite effect, increasing the *T_m_* of AAC2 up to ∼5 °C (Fig. 4, *B* and *C*). This was most prominent in 13M but decreased with the size of the detergent. We observed similar but more severe trends with the substrate ADP. In the presence of phosphatidylcholine or detergent alone, ADP severely destabilized AAC2, particularly in the smaller detergents of those tested, where the *T_m_* dropped to below the lower limit of the assay (Fig. 5, *A* and *B*). These trends would be consistent with the substrate-induced cycling of AAC2 through less stable conformations. However, unlike the effect of bongkrekic acid, higher concentrations of ADP (500 μm) were less destabilizing than lower concentrations (50 μm). In the presence of cardiolipin, however, ADP induced a concentration-dependent stabilization of AAC2 in the medium to large detergents tested (with an increase in *T_m_* of up to 7 °C), with a trend toward a net destabilizing effect in the smaller detergents, particularly at low ADP concentration. Although it is well established that cardiolipin has a role in the c-state of carriers, these trends demonstrate that the lipid also functions in the m-state (and possibly other states), despite the considerable changes that are likely to occur to the interaction sites following state transition (13, 27).

##### Similar Stability Trends Are Observed with UCP1

**FIGURE 6.**
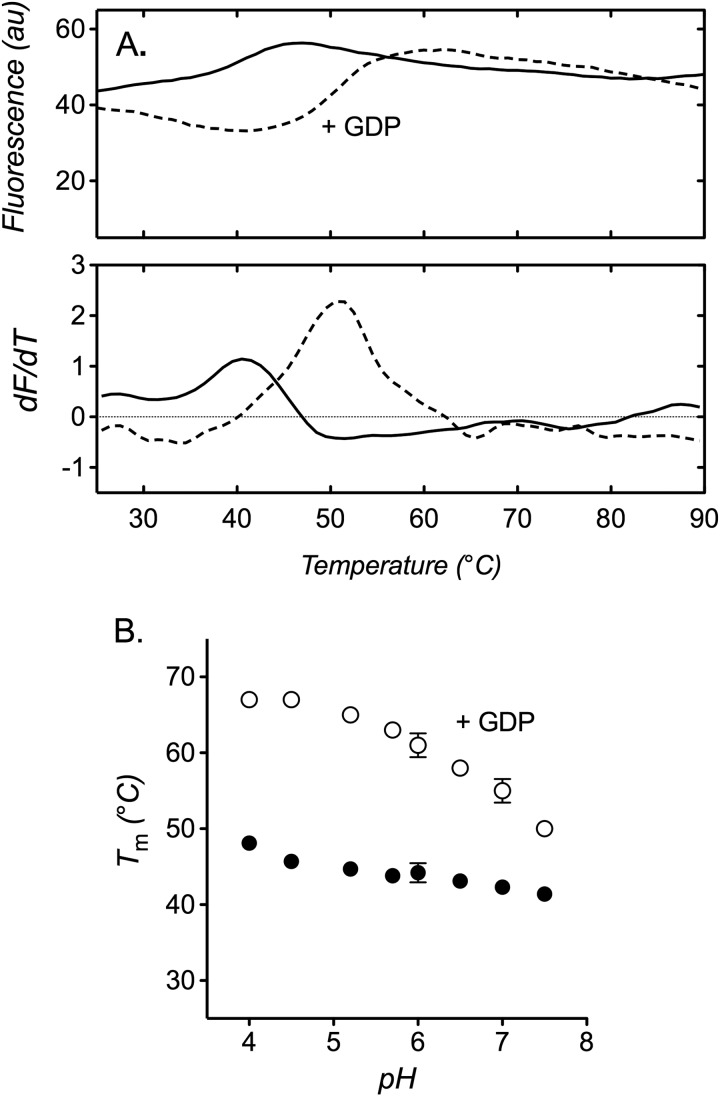
**The thermal stability of native UCP1 and influence of nucleotide binding.** Protein stability assays were performed as described under “Materials and Methods” in assay buffer with 0.1% 10MNG. *A*, thermal denaturation profile (*top*) and corresponding first derivative (*bottom*) of UCP1 in the absence (*solid line*) or presence (*dashed line*) of 2 mm GDP. *B*, change in apparent *T_m_* of UCP1 with pH in the absence (*closed circles*) or presence (*open circles*) of 2 mm GDP. The buffer in the assay was switched to 20 mm Mops, Mes, cacodylate, or gluconate, as required. Typical profiles are shown, and *T_m_* values are averages ± S.D. (*error bars*) of three tests. Ovine UCP1 contains 9 cysteine residues.

We purified UCP1 from native sources in 10MNG detergent (supplemented with cardiolipin) by a novel procedure that, unlike conventional hydroxyapatite methods, allowed the protein to be prepared in defined conditions, free of excess detergent and lipid^4^ (see “Materials and Methods”). In stability measurements, the profile of ovine UCP1 had a raised baseline (*e.g.* see Fig. 6*A*), consistent with evidence that at least one of the nine cysteine residues is accessible in the folded state (44). In 0.1% 10MNG, buffered at pH 7.5, the protein exhibited a relatively low *T_m_* value of 41.4 ± 0.9 °C (Fig. 6*A*), which is comparable with the *T_m_* value of yeast AAC2 in similar conditions (*cf*. Fig. 2). The apparent *T_m_* could be increased to 50.6 ± 0.6 °C by the inhibitor GDP (Fig. 6*A*), consistent with stabilization by ligand binding to the cytoplasmic side of the carrier, as observed with the AAC-CATR complex. The affinity of UCP1 for purine nucleotide inhibitors is known to increase with a decrease in pH, with maximal affinity observed below a pH of ∼6 (28, 45). When assessed at different pH values in buffers free of anions that interfere with nucleotide binding, we found that UCP1 stability reflected this trend. In the presence of GDP, UCP1 showed a large increase in stability toward lower pH values, compared with only a mild trend in the absence of the nucleotide (Fig. 6*B*). At a pH below ∼5, where the influence of tighter GDP binding to UCP1 was maximal, an apparent *T_m_* of ∼67 °C was attained (Fig. 6*B*).

**FIGURE 7.**
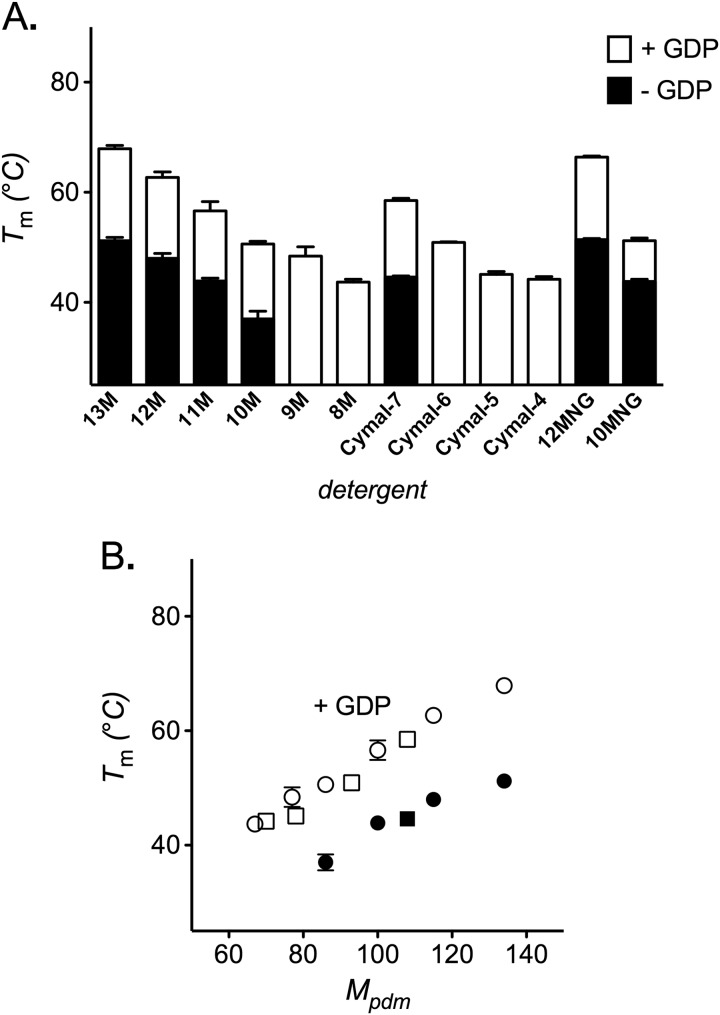
**The influence of detergent on the thermal stability of native UCP1.** Protein stability assays were performed as described under “Materials and Methods” in 20 mm BisTris buffer, pH 6.0, with 1% detergent (2% for 8M) present. *A*, apparent *T_m_* of UCP1 in various detergents in the absence or presence of 2 mm GDP, as indicated. *B*, dependence of UCP1 stability on the mass of the protein-detergent micelle (*M_pdm_*) in the absence (*closed circles*) or presence (*open circles*) of GDP (*M_pdm_* values were taken from Refs. 24 and 25 for yeast AAC3). *T_m_* values are averages ± S.D. (*error bars*) of three tests.

UCP1 exhibited trends similar to those of yeast AAC2 in various detergents (buffered at pH 6.0; Fig. 7 (*cf*. Fig. 2)). In the alkyl maltoside and Cymal series, stability increased with carrier *M_pdm_* (AAC3 mass values (24, 25)) but with a slightly higher apparent *T_m_* than yeast AAC2 at lower *M_pdm_* values (Fig. 7*B*). A similar increase in UCP1 stability with overlying trends in the two detergent series was also observed in the presence of GDP at generally higher *T_m_* values. This trend was more linear than the trend observed with AAC2 in the presence of CATR and revealed only a small variation in the ligand-induced increase in *T_m_* (δ*T_m_*^GDP^) toward higher *M_pdm_* values. The similar trends observed with both UCP1 and AAC2 suggest that the size of the associated detergent micelle is important for the stability of mitochondrial carriers in general.

**FIGURE 8.**
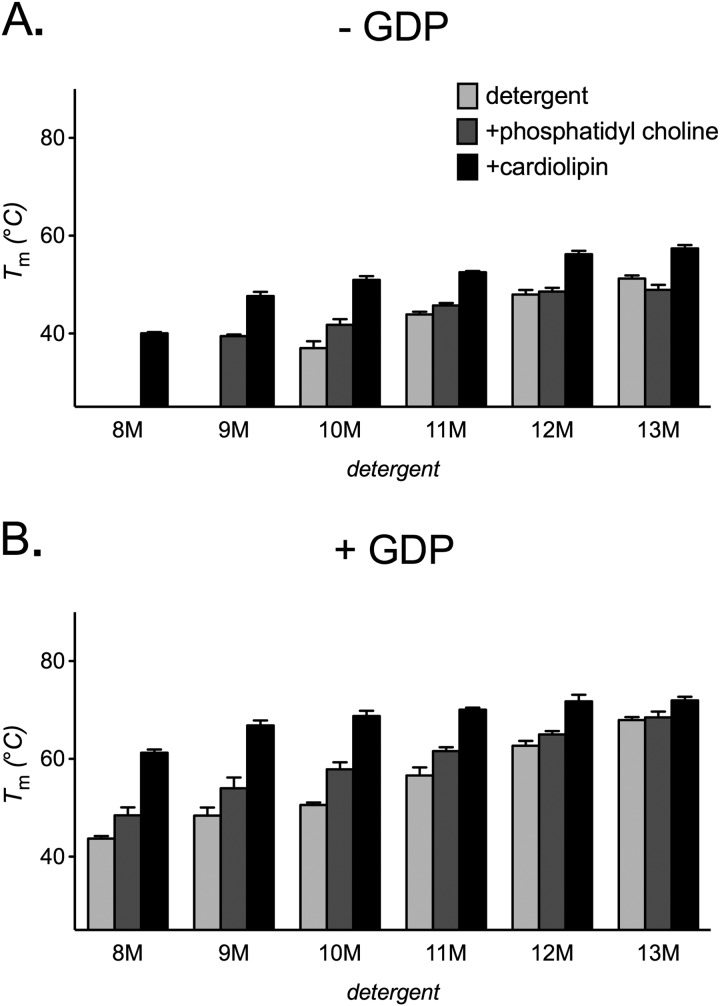
**The influence of lipids on the thermal stability of native UCP1.** Apparent *T_m_* values for UCP1 were determined (see “Materials and Methods”) in 20 mm BisTris buffer, pH 6.0, with 1% detergent (2% for 8M) and 14:0 phosphatidylcholine or cardiolipin (0.1 g/g of detergent) present as indicated, in the absence (*A*) or presence (*B*) of 2 mm GDP. *T_m_* values are averages ± S.D. (*error bars*) of three tests.

In tests with lipids, UCP1 was stabilized by phosphatidylcholine in a manner similar to yeast AAC2. In both the absence and presence of GDP, increases in stability occurred in the smaller alkyl maltoside detergents, where the stability had been lower, with little to no effect in the larger detergents (Fig. 8, *A* and *B*; *cf*. yeast AAC2 (Fig. 3, *A* and *B*)), consistent with lipid-induced stabilization through an increase in the effective detergent micelle size. We have found recently that UCP1 tightly binds cardiolipin, which stabilizes the carrier.^4^ Consistent with this, cardiolipin increased UCP1 stability in *all* of the detergents of the series, including the larger ones. In 12M, for instance, the *T_m_* increased by ∼9 °C in the absence and presence of GDP, although larger increases occurred with smaller detergents, where the initial values had been lower (Fig. 8, *A* and *B*). Like the effect of cardiolipin on the AAC-CATR complex (*cf*. Fig. 3*B*), these trends suggest that cardiolipin stabilizes UCP1 by both micelle size-dependent and -independent mechanisms, even in the absence of GDP ligand. It may be the case that both the GDP-bound and -unbound states of UCP1 are more analogous to the CATR-bound “c-state” of AAC2.

**FIGURE 9.**
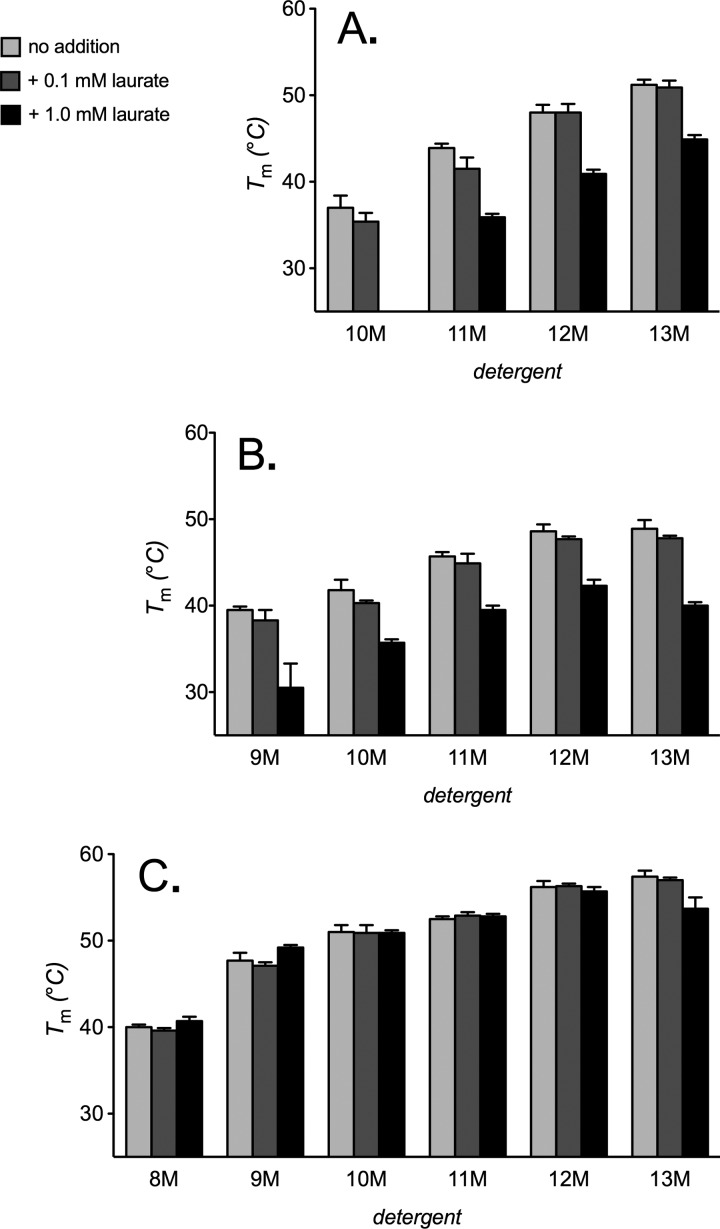
**The influence of lipids on laurate-induced changes in UCP1 thermal stability.** Apparent *T_m_* values for UCP1 were determined (see “Materials and Methods”) in 20 mm BisTris buffer, pH 6.0, with 1% alkyl maltoside detergent alone (*A*) or with 14:0 phosphatidylcholine or cardiolipin (0.1 g/g of detergent) present (*B* and *C*, respectively), with 0.1 or 1.0 mm laurate present where indicated. *T_m_* values are averages ± S.D. (*error bars*) of three tests.

Fatty acid anions activate proton conductance by UCP1 by a disputed mechanism (21, 46). They may act as cofactors, providing protonatable sites within a transport channel of the protein (47), or they may be transport substrates exported by UCP1, returning in a protonated state via the carrier (46) or directly through the mitochondrial inner membrane (48). Alternatively, they may merely act to overcome the binding of inhibitory nucleotides (49). When tested in alkyl maltoside detergents, the apparent *T_m_* of UCP1 was not affected by 0.1 mm laurate but decreased by 6–12 °C with a 1 mm concentration (Fig. 9*A*). This effect was similar in the presence of phosphatidylcholine but was not observed in the presence of cardiolipin (Fig. 9, *B* and *C*, respectively). By analogy with the cardiolipin-sensitive influence of ADP and bongkrekic acid on AAC2, these trends would suggest that laurate has a substrate-like interaction with UCP1, shifting the protein to a less stable state.

**FIGURE 10.**
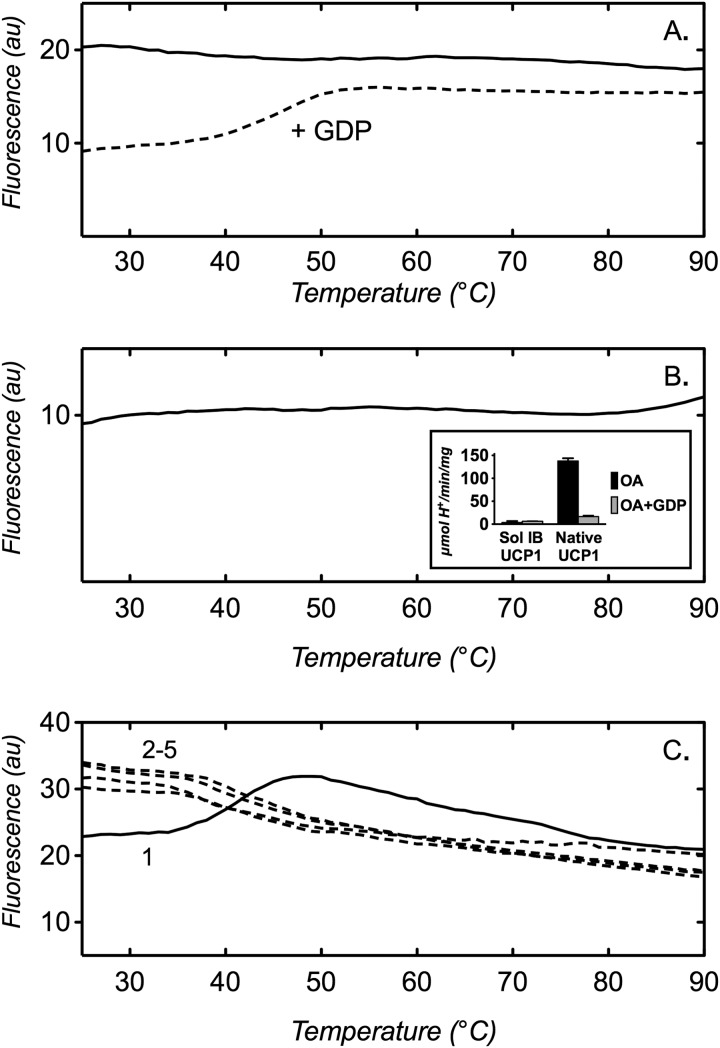
**UCP1 can appear soluble but is unfolded in some conditions.** Protein stability assays were performed as described under “Materials and Methods.” Thermal denaturation profiles are as follows. *A*, native UCP1 in 0.2% Fos12 in the absence (*solid line*) or presence (*dashed line*) of 2 mm GDP; *B*, Fos12 (DPC)-solubilized mouse UCP1 inclusion bodies in 0.1% Fos12 assay buffer (*inset*, corresponding oleic acid (*OA*)-dependent proton conductance activity of the preparation compared with native UCP1 controls after reconstitution into liposomes (see “Materials and Methods”)); *C*, native UCP1 in 0.1% 10MNG assay buffer run initially (*solid line*, *1*) and following cooling and retesting of the same sample in 2–5 consecutive assay runs (*dashed lines*, runs *2–5*, respectively). The assay buffer was composed of 20 mm Mes, pH 6.5 (*A* and *C*), or 20 mm Hepes, pH 7.5 (*B*). Typical profiles are shown. *Error bars*, S.D. of three tests.

The detergent Fos12 (DPC) has been used to purify *E. coli* expressed UCP1 and UCP2 proteins and provide material for the structural determination of UCP2 by novel NMR methods (31). However, scrutiny of the UCP2 structure in molecular dynamic simulations and tests with reconstituted UCP1 suggest that the structural integrity of carriers is likely to be compromised in this detergent (29). In direct measurements of stability, we found that native UCP1 was relatively unstable in 0.2% Fos12 (buffered at pH 6.5; Fig. 10*A*), with an apparent *T_m_* of <25 °C in the absence of GDP (*cf*. 43.1 ± 1.0 °C in 0.1% 10MNG; Fig. 6*B*) and 44.9 ± 2.3 °C in the presence of GDP (*cf*. 58.0 ± 0.2 °C in 0.1% 10MNG; Fig. 6*B*). Furthermore, this detergent proved to be harsh enough to solubilize UCP1 inclusion body aggregates, which gave a stability profile with a maximal signal across all temperatures with no unfolding transition, indicative of protein already in an unfolded state (Fig. 10*B*). In agreement with this, no UCP1-like activity was observed following the reconstitution of this preparation into liposomes, in contrast to native UCP1 controls (Fig. 10*B*, *inset*). Interestingly, we found that mitochondrial carriers appeared to remain soluble in mild detergents after thermal denaturation. Samples that were unfolded from a stability assessment, cooled, and retested, showed profiles with a maximal signal that did not decrease in consecutive assay runs (*e.g.* see Fig. 10*C*), suggesting that the protein was not aggregating and falling out of solution. Apparent solubility, therefore, even in mild detergents, is not necessarily a marker of a suitably folded carrier.

## DISCUSSION

Membrane proteins, particularly from eukaryotic organisms, are typically unstable when purified in detergents, which presents a major limitation to structural and mechanistic studies (50). The assessment of protein stability is vital to confirm sample integrity and optimize stabilizing conditions. Here, we have adapted a fluorescence-based thermostability assay (32) applicable to membrane proteins for use on a rotary qPCR multisample instrument, to give a rapid and high throughput procedure, using relatively small amounts of protein. With this setup, we have assessed the stability of various mitochondrial carrier proteins in detergents. As well as highlighting optimal conditions, the detailed trends in stability reveal mechanistic insights into carrier interactions with substrate, lipids, and state-specific inhibitors.

We demonstrate that the stability of mitochondrial carriers in non-ionic detergents increases with the size of the associated detergent micelle. Membrane proteins often exhibit a preference for larger non-ionic detergents to allow purification, and similar trends can be observed in stability data for other membrane proteins too (*e.g.* the APJ receptor (32)). Larger micelles will have increased interaction energy due to the additional molecular interactions associated with the extra mass, which may restrain the protein more during thermal motions. We found that the conjugated maltose-neopentyl glycol class of detergents stabilized mitochondrial carriers more than the unconjugated alkyl maltoside equivalents, as observed for other membrane proteins (51). The higher stability most likely relates to an increase in the total interaction energy of the micelle due to the additional covalent bonds in the detergent (rather than from an increase in micelle size). Our stability trends also suggest that lipids in general stabilize mitochondrial carriers indirectly by increasing the size of the associated detergent micelle. Lipids have some similar features to detergents but typically have longer alkyl chains and so, at sufficient concentrations, will distribute within a detergent micelle and increase the average micelle size and thus the stability.

As well as increasing the micelle size, cardiolipin has specific interactions with mitochondrial carriers, in agreement with the observation that it affects their function (see Ref. 27 and references therein). Cardiolipin binds tightly to AAC in complex with CATR (52), as observed in the bovine and yeast structures (7, 12, 13). Importantly, our results show that bongkrekic acid or ADP destabilize AAC2 in the absence of cardiolipin yet stabilize the protein in the presence of the lipid, which reveals that cardiolipin interacts with the m-state and other states relevant to substrate turnover. Analogous to this, fatty acid activators destabilize UCP1 in a similar manner, but not in the presence of cardiolipin, indicating that they act like substrates in the mechanism of proton conductance. This is consistent with a fatty acid-induced conformational change in the protein, as suggested by others (53). Accordingly, our observations would, therefore, favor the mechanistic models of proton transfer by UCP1 that rely on fatty acid/fatty acid anion transport (see Refs. 46 and 48). Our data also show that cardiolipin stabilizes the GDP-free and -bound states of UCP1, consistent with our recent finding that it binds tightly to this protein in a manner similar to AAC.^4^ In contrast to fatty acids, GDP significantly stabilized UCP1, particularly at low pH, where the protein is known to have increased affinity for nucleotides (28, 45). Purine nucleotides are physiologically relevant regulators of UCP1 proton conductance and would appear to shift the protein into a “tight” inhibited state (54). All of these observations would be in agreement with our proposal that cardiolipin functions to protect a dynamic interface between mobile domains of the carrier during the transport cycle (13).

We show that our approach can unambiguously determine whether a protein in detergent solution is folded or not. We found that carriers exhibited particularly poor stability in the zwitterionic detergent Fos12, which proved harsh enough to solubilize UCP1 inclusion body material and maintain the unfolded protein in a soluble state. These observations, along with those of others (29), raise serious concerns about the structural integrity of carriers prepared in this type of detergent (*e.g.* see Refs. 30 and 31), which was used in determining a backbone structure of recombinant UCP2 by a new NMR approach (31). In the absence of the stabilizing effects of tight ligand binding at nanomolar potency (*e.g.* the AAC-CATR complex (7)), any correctly folded UCP2 in Fos12 is unlikely to be stable, particularly in NMR experiments at 33 °C (31). We found that unfolded UCP1 can appear soluble in non-ionic detergent as well, which further emphasizes the need for robust methods to confirm a native carrier fold. This is particularly important for mitochondrial carriers expressed in *E. coli*, which typically require “refolding” from unfolded inclusion body material. Unfortunately, the common methods used to verify recombinant proteins may give artifactual results that can be interpreted to suggest the presence of folded carrier, particularly in the absence of control protein from native sources. For instance, fluorescence resonance energy transfer (FRET) from UV light-excited UCP material to nucleotide-conjugated fluorophores, such as mant-GDP or -ATP (31, 55), can easily arise through nonspecific interactions, and the signal dampening that is ascribed to competition from unconjugated nucleotide may merely be a consequence of the reduced excitation of the whole system due to the strong absorbance of UV excitation light by nucleotides. Similarly, decreases in UCP tryptophan fluorescence (30, 56) in the presence of nucleotides may reflect nonspecific interactions or relate to nucleotide UV light absorption and so are somewhat arbitrary in the absence of native protein controls. The α-helix signatures in CD spectra of UCP preparations are also poor indicators of correctly folded carriers because secondary structure may be retained without tertiary structure. Our controls with native UCP1 indicate that strong α-helix profiles, with shape and amplitudes comparable with those of recombinant UCP preparations (30, 56–58), can be obtained with UCP1 denatured in SDS.^4^ In other methods, the apparent activity of “refolded” carriers reconstituted into proteoliposomes (30, 31, 59) may only relate to a small proportion of correctly folded material, which could be generated in the liposome formation procedure itself. Importantly, our results demonstrate that a simple and economical thermostability assay can be used to validate folded carrier protein and confirm important characteristics of the native protein from unique trends in stability.

## Footnotes

4 Y. Lee, C. Willers, E. R. S. Kunji, and P. G. Crichton, manuscript in preparation.

## Footnotes

3 The abbreviations used are:

CATR: carboxyatractyloside
CPM: *N*-[4-(7-diethylamino-4-methyl-3-coumarinyl)phenyl]maleimide
8M: octyl-β-d-maltoside
9M: nonyl-β-d-maltoside
10M: decyl-β-d-maltoside
11M: undecyl-β-d-maltoside
12M: dodecyl-β-d-maltoside
13M: tridecyl-β-d-maltoside
10MNG and 12MNG: 2,2-dioctylpropane- and 2,2-didecylpropane-1,3-bis-β-d-maltopyranoside, respectively
LAPAO: 3-laurylamido-*N*,*N*′-dimethylpropyl amine oxide
Fos12 or DPC: Fos-choline-12 (*n*-dodecylphosphocholine)
qPCR: quantitative PCR
HRM: high resolution melt
BisTris: 2-[bis(2-hydroxyethyl)amino]-2-(hydroxymethyl)propane-1,3-diol
mant: *N*-methylanthraniloyl.
